# Synthesis of new Cr_2_O_3_/Fe_2_O_3_/glass composites from industrial wastes; from undesired to advanced optical products

**DOI:** 10.1007/s11356-022-21694-w

**Published:** 2022-07-02

**Authors:** Dina H. A. Besisa, Hanan H. Mohamed, Emad M. M. Ewais, Yasser M. Z. Ahmed, Amira M. M. Amin

**Affiliations:** 1 Refractory & Ceramic Materials Division (RCMD), Central Metallurgical R&D Institute (CMRDI), P.O. Box 87, Helwan, Cairo 11421 Egypt; 2 Faculty of Science, Chemistry Department, Helwan University, Cairo, 11795 Egypt

**Keywords:** LSG wastes, Cr_2_O_3_/Fe_2_O_3_ ceramics, Microstructure, Optical properties, Magnetic properties

## Abstract

For the tendency toward cleaner production and safe conversion of undesired toxic wastes to highly priced advanced products, this work introduces new ceramics/glass composites of Cr_2_O_3_/Fe_2_O_3_/lead silicate glass (LSG) from industrial LSG wastes. Both chromia Cr_2_O_3_ and hematite Fe_2_O_3_ ceramics are added equally to the LSG wastes with different percentages (10, 20, and 30 wt.%) via the pressureless sintering method. The competitiveness of this work is dependent on the conversion of undesired waste materials into advanced/smart optical materials with a low cost and an environmentally friendly method. Hence, the influence of both Cr_2_O_3_ and Fe_2_O_3_ additions on the behavior and the different characteristics of the lead silicate wastes are comprehensively investigated. Evaluation of the final ceramics/glass composites was achieved through their phase composition, microstructure, optical, and magnetic characteristics. The results verified that the insertion of both chromia and hematite together into the glass waste had a key role in improving its morphological properties and optical and magnetic behaviors. Composite with 30% of Cr_2_O_3_/Fe_2_O_3_ gave the highest optical absorbance of 90%, the lowest and best band gap energy of 1.68 ev, and the highest refractive index of 2.85. Also, it recorded the best magnetic behavior with the highest saturation magnetization of 139.700 × 10^−2^A m^2^ kg^−1^ and the best coercivity of 190.0 Oe. These findings confirmed the successful clean conversion of the hazardous lead silicate waste into advanced products with promising optoelectronic characteristics.

**Graphical abstract:**

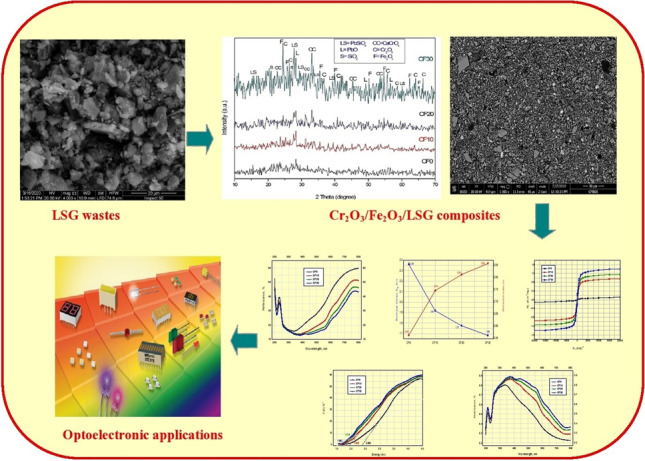

## Introduction

Rapid global manufacturing has not only led to the development and civilization of countries but also to the generation of hazardous industrial waste in huge amounts. Solid industrial wastes generated from the industrial activities of the different chemical and developed plants cause harmful environmental pollution (Hoornweg and Bhada-Tata 2012). Management and recycling of these wastes are considered a serious challenge and inadequate due to the lack of integrated/sustainable management plans and insufficient resources.

Industrial LSG wastes discarded by glass and crystal manufacturing companies, in particular, were classified as extremely hazardous materials (Besisa et al. 2021; Ishchenko 2019). The severity of these glass wastes comes from the presence of a high concentration (30%) of toxic lead compounds (Meng et al. 2016). So, in order to get rid of these undesirable materials, there is a need for new applications.

Various investigations have included the recycling of the lead silicate waste revealed from the cathode ray tube (Meng et al. 2016; Fernandes et al. 2021; Guo et al. 2010a and b). However, very few studies have been conducted on the same wastes released by glass manufacturing companies (ElKersh and El-Haggar 2015; Zhernovaya and Onishchuk 2005). Besides, these studies failed to recognize the exact effect of the lead compounds on the glass properties. They could not successfully convert them into advanced products for new applications.

Furthermore, it was reported that combining glass and ceramic materials together in one composite structure results in a new polycrystalline material with desirable properties and tailored applications (Ahmed et al. 2017; Besisa and Ewais 2019). The type and properties of the inserted ceramic materials can determine the final characteristics and the new applications of the original glass (Sandu et al. 2017a, b). For example, Cr_2_O_3_ was added to iron phosphate glass to enhance the optical properties of the parent glass. The obtained glass/ceramic composite was nominated as a highly efficient luminescence material (Šantic et al. 2007). Another interesting thing about this glass/ceramic system is its low processing temperatures compared to those required for engineering ceramic materials.

On the other hand, hematite (α-Fe_2_O_3_) and chromia (Cr_2_O_3_) are isostructural ceramic oxides with a corundum structure. They form a continuous and homogenous solid solution in the entire concentration range (Grygar et al. 2003). It was reported that the combination of hematite and chromia has great importance in many technological and industrial applications. Together, they can exhibit outstanding optical, magnetic, and catalytic properties. This behavior arose from the attractive characteristics of both Cr_2_O_3_ and Fe_2_O_3_. Chromia has been considered a p-type semiconductor with wide band gap energy. It has high electrical conductivity, high thermal and optical properties, thermal stability, and high temperature oxidation resistance (Cao et al. 2006; Hammoudeh et al. 2006; Santulli et al. 2011). Also, hematite is a well-known optical/magnetic material with promised photocatalytic behavior (Ohkoshi et al. 2016; Besisa and Ewais 2021). It has a remarkable effect on improving the physical properties of the original glass materials (Ibrahim et al. 2014).

Hence, it can be believed that the addition of Cr_2_O_3_ and Fe_2_O_3_ together with varying their compositions to glass materials can produce new ceramic/glass composites with homogenous solid solution/diffusion reaction and promised performance/application. To the best of our knowledge, no work has investigated the addition of chromia and hematite together to lead silicate glass before or investigated its characteristics neither from a pure source nor in waste form.

In this work, we have two challenges to overcome and achieve. The first one is finding a cheap radical environmental solution for the disposal of the accumulated lead silicate glass waste. The second one is producing advanced glass/ceramic materials for new optical and magnetic applications from these wastes. Obtaining this novel material will lead to produce a sustainable high-performance product suitable for new advanced applications. These can be attained by the addition of Cr_2_O_3_ and Fe_2_O_3_ together with different compositions to the revealed LSG waste by using the pressureless sintering method. Moreover, different properties of the produced glass/ceramic composites will be evaluated, such as physical, morphological, magnetic, and optical properties.

## Experimental

### Materials and preparation

The parent glass material used in this investigation is the solid waste of lead silicate crystal glass production at the final polishing and finishing stage. It was supported by Asfour Crystal manufacturing company, Shoubra, Egypt. Details of the solid lead silicate glass wastes processing, as well as its chemical and physical analysis, are described in detail (Besisa et al. 2021). Highly pure (99.999%) Fe_2_O_3_ and Cr_2_O_3_ were added equally with different contents to the parent glass in order to create various glass/ceramics composites. Raw Fe_2_O_3_ and Cr_2_O_3_ were provided by Loba Chemie Pvt. Ltd., India. Four Fe_2_O_3_/Cr_2_O_3_/LSG composites with various content of Fe_2_O_3_/Cr_2_O_3_ (0, 10. 20, and 30 wt%) were obtained. Composite nomenclature with different additions of Fe_2_O_3_, Cr_2_O_3_, and the lead silicate waste is demonstrated in Table 1. Composite mixtures were uniformly blended by a planetary ball mill for 2 hrs in 100% ethanol with zirconia balls. Green compacts in cylindrical form with dimensions of 13.7 mm in diameter were produced by uniaxial cold pressing (at ≈ 400 MPa). The different sintered Fe_2_O_3_/Cr_2_O_3_/lead silicate glass composites were obtained by pressureless sintering at a temperature of 600 °C/2 h of the green compacts with a heating rate of 5 °C/min.

**Table 1:** The designation (wt%)/nomenclature of the various Fe_2_O_3_/Cr_2_O_3_/LSG composites

Sample	Lead silicate glass waste LSG (wt%)	Fe_2_O_3_ (wt%)	Cr_2_O_3_ (wt%)
CF0	100	0	0
CF10	90	5	5
CF20	80	10	10
CF30	70	15	15

### Characterization

Phases identification of the waste and the produced composites are recognized by a Bruker D8-advance X-ray powder diffractometer with Cu Ka radiation (*k* = 1.5406 Å).

Microstructural properties of the obtained composites are examined by using field emission scanning electron microscopy (FESEM; QUANTA FEG250, Holland) connected to an energy-dispersive X-ray microanalyzer (EDX).

Optical absorbance and reflectance of the sintered composites are measured by using a UV-VIS spectrophotometer (Jasco-V-570 spectrophotometer, Japan) fitted with an integrating sphere reflectance unit (ISN) in the wavelength range of 200–800 nm.

Magnetic properties are measured using a vibrating sample magnetometer (VSM) Lake Shore, USA.

## Results and discussion

### Phase analysis and microstructure examination

**Fig. 1:**
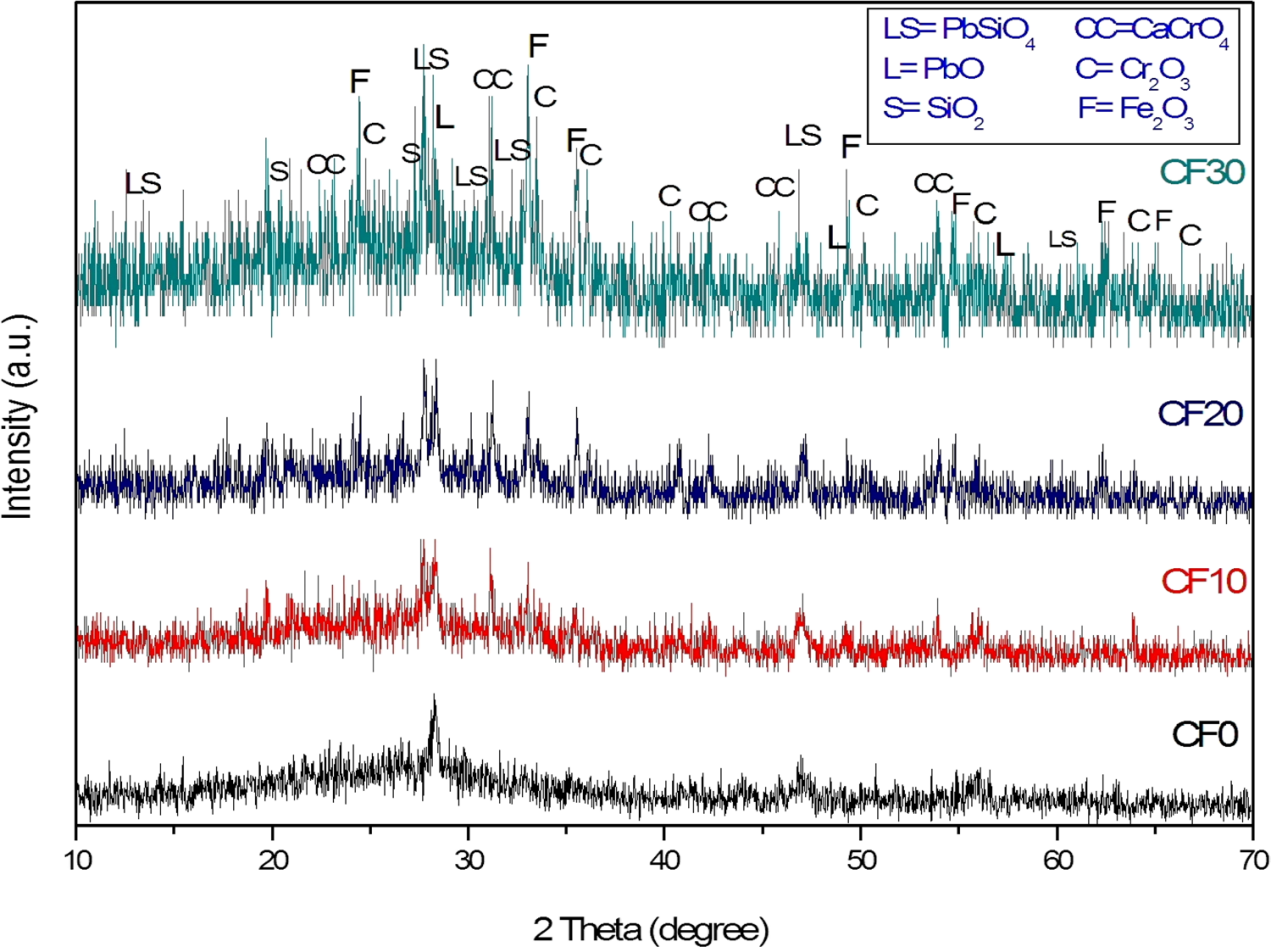
XRD pattern of the different sintered Cr_2_O_3_/Fe_2_O_3_/LSG composites

The XRD patterns of the sintered Cr_2_O_3_/Fe_2_O_3_/LSG composites with various Cr_2_O_3_+ Fe_2_O_3_ content (0–30 wt%) are shown in Fig. 1. It was revealed that adding and increasing the wt% of Cr_2_O_3_ and Fe_2_O_3_ has led to enhancing the crystallinity of the parent LSG material. It resulted in the formation of highly crystalline Cr_2_O_3_/Fe_2_O_3_/LSG composites, especially for CF30 composite. In another sense, adding and increasing the content of ceramic materials has transformed the amorphous glass structure into a polycrystalline composite material. For sintered composite without chromia and hematite addition (CF0), the main formed phases were lead silicate (Pb_2_SiO_4_), lead oxide (PbO), calcium silicate (Ca_2_SiO_4_), and silica (SiO_2_). Calcium silicate was formed as the result of a direct reaction between calcium carbonate and silica that was found in the parent glass waste (Kale et al. 2012).

However, upon the addition of 10% Cr_2_O_3_+ Fe_2_O_3_ (CF10 composite), the formed calcium silicate was substituted by calcium chromium oxide (CaCrO_4_). This behavior was attributed to the fact that the reactivity of added chromia was higher than silica. Hence, part of the added chromia diffused through the formed calcium silicate, substituted the silicate position, and formed a solid solution of calcium chromium oxide. The other part existed as a single chromia phase. With increasing chromia and hematite content to 20 and 30%, the crystallinity and content of the formed chromia, calcium chromium oxide, and hematite increased and enhanced, which confirms the successful conversion of the parent glass to polycrystalline ceramic composites. The more interesting thing is that CaCrO_4_ has outstanding optical, catalytic, and photoluminescent properties. It has been widely used in various technological applications such as photosensitization, catalysis, scintillation, photoluminescence, pigments, and photoconductive dielectric materials (Driscoll and Ozkan 1994; Kobayashi et al. 1998; Yu et al. 2002; Liu et al. 2004; Xiang et al. 2004). Hence, it is expected to have a significant effect on the optical performance of the investigated glass/ceramic composites.

**Fig. 2:**
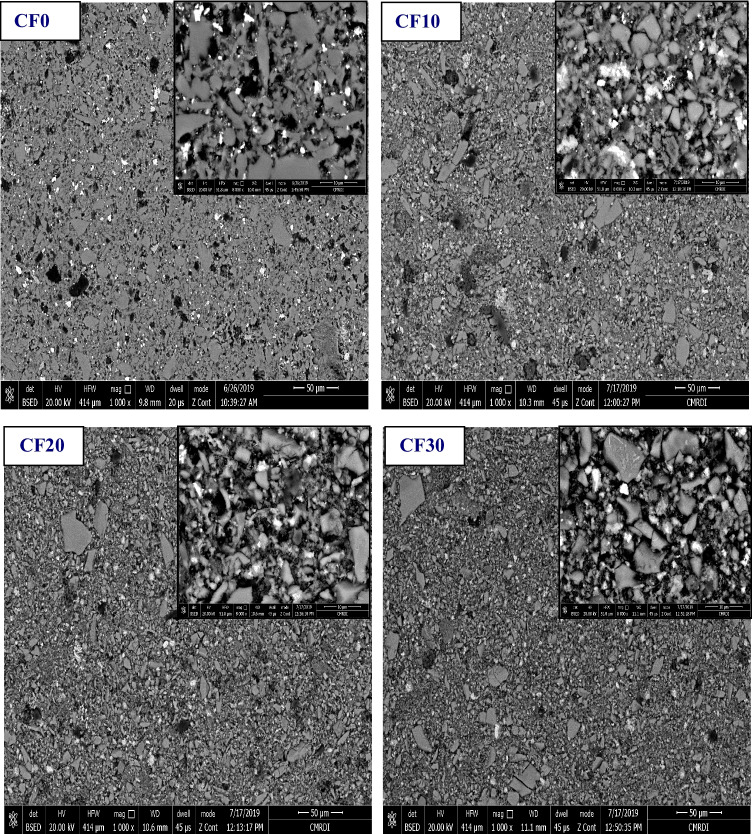
Microstructure of the different sintered Cr_2_O_3_/Fe_2_O_3_/LSG composites

The microstructure of the obtained ceramics/glass composites is illustrated in Fig. 2. It was obviously observed that the grain surface and edges of the CF0 sample without the addition of chromia or hematite are inconspicuous due to the amorphous nature of the parent LSG. However, addition of Cr_2_O_3_ and Fe_2_O_3_ has enhanced the appearance, homogeneity, and topographic features of all the formed phases through the different sintered ceramic/glass composites. Also, there was a variety in the grain size and morphology of the formed phases. This diversity in the grains shapes between cubic, elongated, and angular structures can be related to the formation of new phases and also to the intimate diffusion between the glass phases and the added chromia+hematite phases in each composite.

### Optical properties

**Fig. 3:**
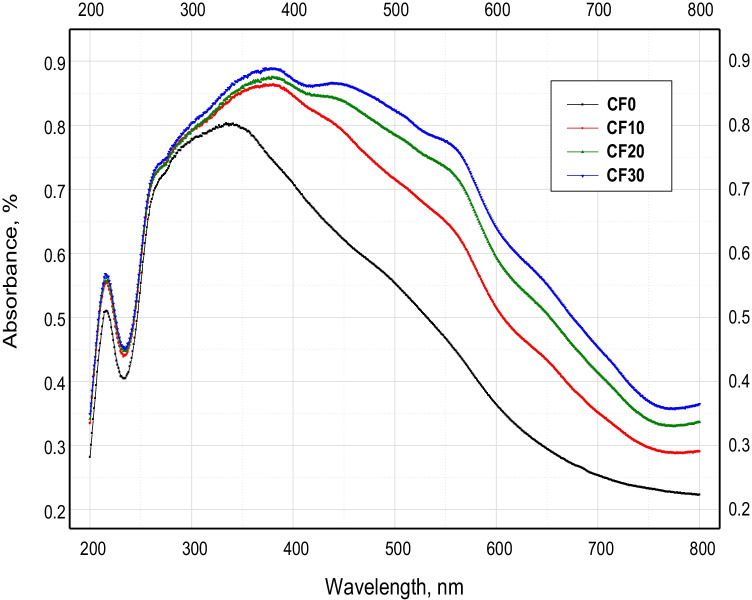
UV-VIS absorbance spectrum of the different sintered Cr_2_O_3_/Fe_2_O_3_/LSG composites

**Fig. 4:**
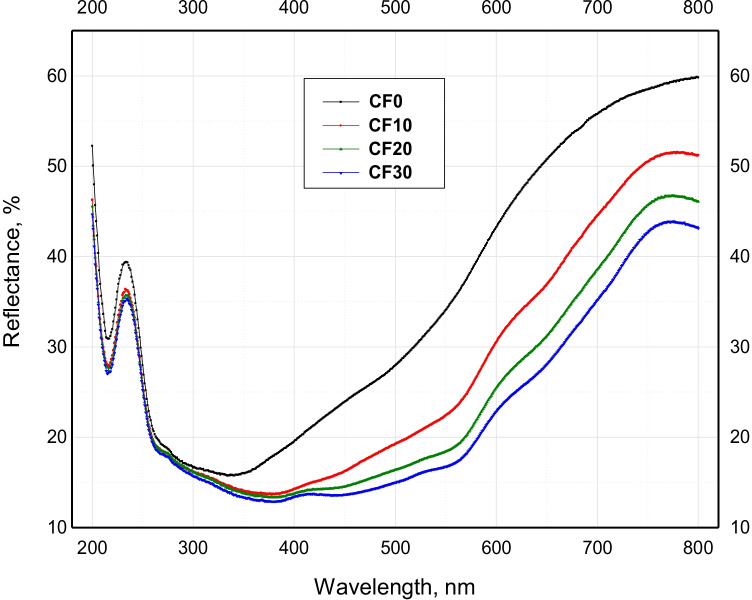
UV-VIS reflectance spectrum of the different sintered Cr_2_O_3_/Fe_2_O_3_/LSG composites

The UV-Visible absorption and reflectance spectra of the different sintered Cr_2_O_3_/Fe_2_O_3_**/**LSG composites are depicted in Figs. 3 and 4. Noticeably, the various obtained composites from CF0 to CF30 have exhibited absorption and reflectance in the whole investigated light range (200–800 nm). Also, it was confirmed that increasing the ceramic materials content has a significant impact on promoting the optical absorbance and reflectance. In other words, CF30 composite with the highest percentage of Cr_2_O_3_+ Fe_2_O_3_ (30%) had the best absorbance of almost 90% in the visible light region. However, LSG material without the addition of chromia and hematite (CF0) recorded the best absorbance of 79% in the UV light region only at a wavelength of ≈ 330 nm (Fig. 3). This glass material gave only two absorption bands in the UV light range: one sharp with a maximum at a wavelength of 215 nm and the other is a broad one with a spectral wavelength range of 260–380 nm. However, upon the addition of Cr_2_O_3_ and Fe_2_O_3_, the second broad band at 260–380 nm was moved toward the visible light range, with a spectral wavelength range of 260–420 nm. Besides, new broad absorption bands were observed at the wavelength ranges of 420–530 nm and 530–600 nm. The release of these strong and enhanced absorption bands resulted from the indirect charge transition of Fe^3+^ 3d→3d (visible absorption) and the direct charge transition of O_2_^−^ 2p→Fe^3+^ 3d (UV absorption) (Zhang et al. 2010; Cao et al. 2006). Absorption in the range of 260–420 nm is caused by the d–d transition in Cr^3+^ (3d^3^) of Cr_2_O_3_ (Mirhadi and Mehdikhania 2011). Furthermore, absorption bands in the wavelength range of 530–600 nm refer to the peaks of Cr^3+^ ion in glass and calcium chromate. This behavior confirms the functional role of chromia and hematite in promoting the optical performance of the parent lead silicate glass material.

On the other hand, it was found that smart materials used in optical applications should have high optical performance. Thus, the band gap energy and refractive index of the various sintered Cr_2_O_3_/Fe_2_O_3_/LSG composites are detected. The band gap energy (Eg) of the sintered composites was calculated according to the Kubelka–Munk function (Feng et al. 2018).

**Fig. 5:**
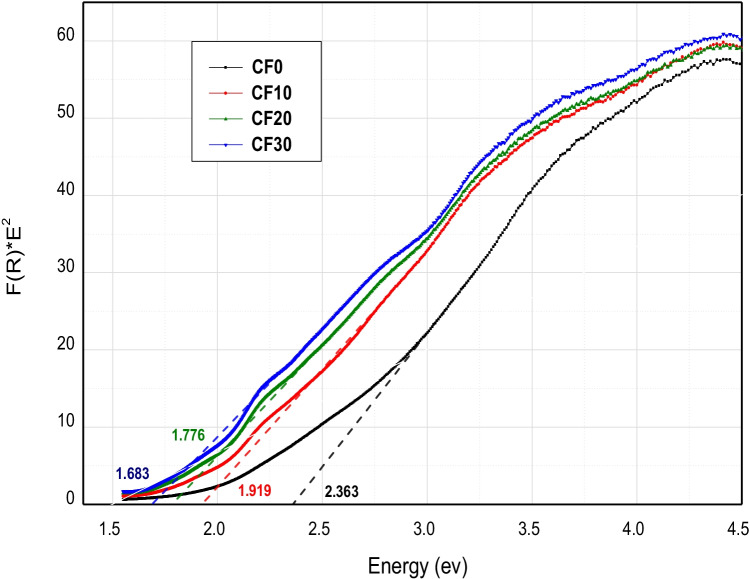
Band gap energy of the different Cr_2_O_3_/Fe_2_O_3_/LSG composites

The optical band gap energy of the different Cr_2_O_3_/Fe_2_O_3_/LSG composites is shown in Fig. 5. Obviously, the band gap energy of the obtained Cr_2_O_3_/Fe_2_O_3_/LSG composites decreased with increasing Cr_2_O_3_+Fe_2_O_3_ content. Its value decreased from 2.363 ev for CF0 composite until it reached its lowest and best value of 1.68 ev for CF30 composite with the 30% addition of Cr_2_O_3_+Fe_2_O_3_. This enhanced behavior can be explained according to the fact that Cr_2_O_3_ and Fe_2_O_3_ acted as dopants inside the glass structure and led to the generation of new discrete energy levels between its valence and conduction band. Accordingly, they will decrease the transition energy of the transferred electron, and, at the same time, they will act as electron (or hole) traps (Mardare and Hones 1999).

**Fig. 6:**
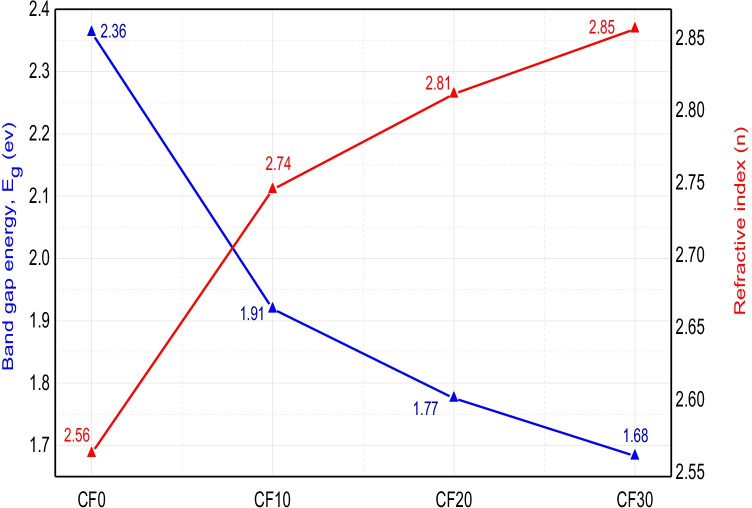
Variation of refractive index with calculated band gap values of the sintered Cr_2_O_3_/Fe_2_O_3_/LSG composites

High refractive index is a characteristic of high light dispersion efficiency and hence high optical performance. The refractive index (n) of the pressureless sintered composites is estimated according to Herve and Vandamme’s relation (Herve and Vandamme 1994):

$${\mathbf{n}}^{\boldsymbol{2}}=\mathbf{1}+{\left[\mathbf{A}/\left({\mathbf{E}}_{\mathbf{g}}+{}_{\mathbf{B}}\right)\right]}^{\mathbf{2}}$$

where *A* and *B* are constants with values of 13.6 eV and 3.4 eV, respectively. *E*_*g*_ is the optical band gap energy value. Figure 6 illustrates the relationship between the calculated refractive index and band gap energy values of the obtained Cr_2_O_3_/Fe_2_O_3_/LSG composites. Refractive index values were found to be mainly dependent on the added ceramic material content. Meaningly, increasing the weight percent of the added chromia and hematite to 30% has a significant role in enhancing the refractive index of the original waste material. It gave the highest refractive index value of 2.85. So, we can conclude that ceramics/glass composite with ceramics content of 30 wt% is a promising candidate in optical and optoelectronic applications.

### Magnetic properties

**Fig. 7:**
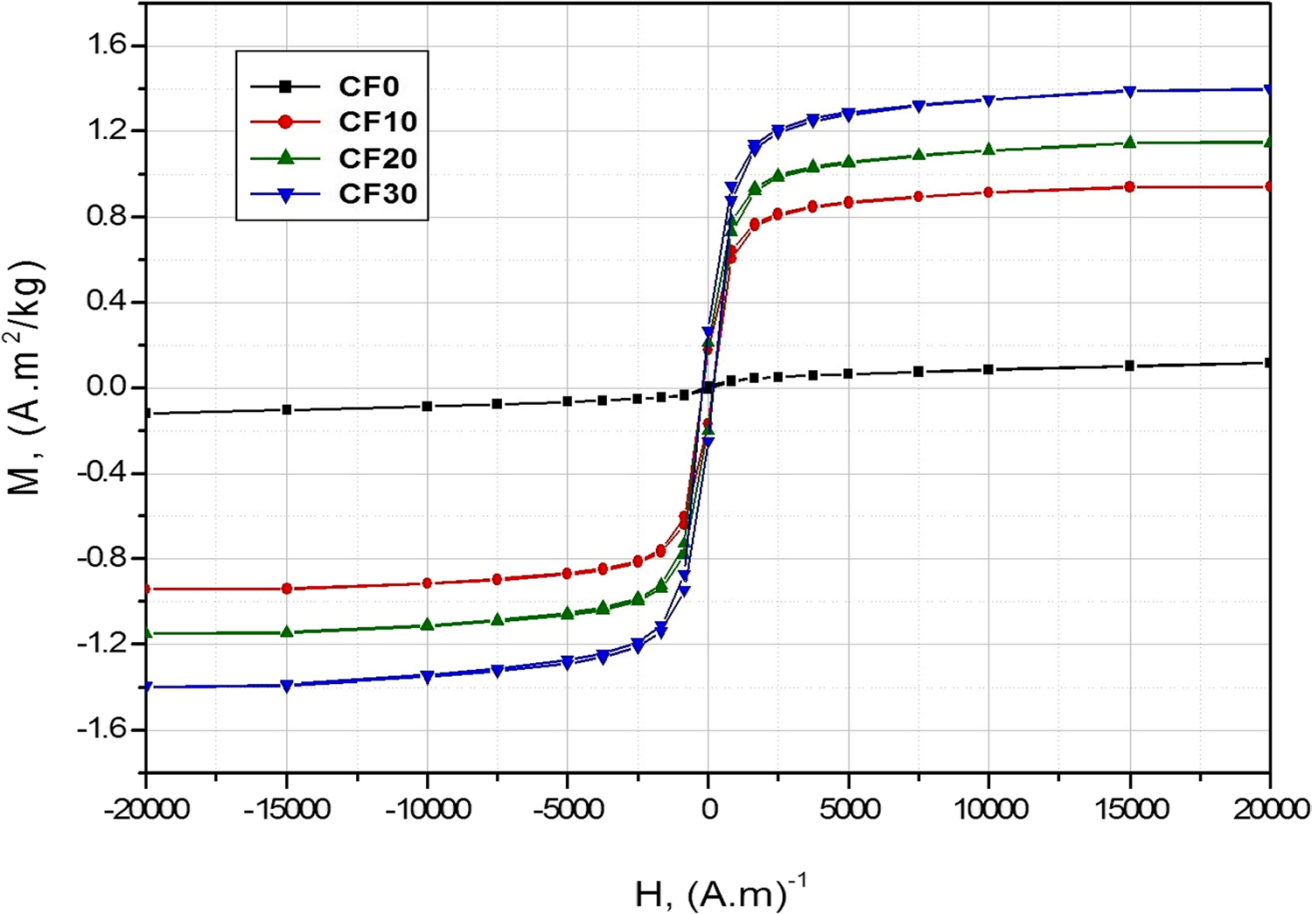
Magnetic curve of the sintered Cr_2_O_3_/Fe_2_O_3_/LSG composites

Functional magnetic materials are playing a major role in various technological uses such as energy storage, microelectronics, biomedical, and optical applications (Matzui et al. 2019; Kozlovskiy et al. 2021). Specifically, some tailored glass/ceramic materials with high magnetic characteristics have already been used in wide industrial applications (Ferreira et al. 2021; Deng et al. 2019; Worsch et al. 2013). Accordingly, the magnetic properties of our proposed Cr_2_O_3_/Fe_2_O_3_/LSG composites are investigated at room temperature according to Fig. 7 and Table 2.

**Table 2:** Magnetic parameters of the different sintered Cr_2_O_3_/Fe_2_O_3_/LSG composites

Sample	M_s_, A m^2^ kg^−1^	M_r_, A m^2^ kg^−1^	H_c_, (Oe)
CF0	11.694 × 10^−2^	7.0498 × 10^−3^	149.37
CF10	94.242 × 10^−2^	173.19 × 10^−3^	183.10
CF20	114.970 × 10^−2^	205.38 × 10^−3^	185.80
CF30	139.700 × 10^−2^	258.55 × 10^−3^	190.01

The magnetic behavior of the pressureless sintered Cr_2_O_3_/Fe_2_O_3_/LSG composites is shown in Fig. 7. Moreover, the measured magnetic parameters such as saturation magnetization (M_s_), remanent magnetization (M_r_), and coercive field (H_c_) are listed in Table 2. Noticeably, the original glass material without ceramic addition (CF0 sample) shows very weak magnetic behavior. However, adding and increasing the ceramics content of Cr_2_O_3_ and Fe_2_O_3_ had an unparalleled influence on promoting the magnetic behavior of the obtained composites. In other words, Cr_2_O_3_/Fe_2_O_3_/LSG composites with 10 to 30 wt% addition of Cr_2_O_3_+Fe_2_O_3_ exhibited strong ferromagnetic behavior with improved saturation magnetization. The hysteresis loop area is obviously increased with increasing the wt% of Cr_2_O_3_+Fe_2_O_3_. The best magnetic behavior with the highest magnetization was recorded by the 30% ceramics/glass composite (Table 2). For example, the addition of 10% Cr_2_O_3_+Fe_2_O_3_ to the original glass waste resulted in boosting the saturation magnetization from 11.694 × 10^−2^ to 94.242× 10^−2^A m^2^ kg^−1^ and the coercivity from 149.3 to 183.1 Oe for CF0 and CF10 composites, respectively. Furthermore, increasing the ceramic addition to 30% (CF30 sample) has pointedly increased the saturation magnetization to 139.700 × 10^−2^A m^2^ kg^−1^ and the coercivity to 190.0 Oe. This promotion and improvement in the magnetic properties of the obtained composites is explained by increasing the concentration of the added ferromagnetic ceramic phases to the original glass waste material (Sandu et al. 2017a, b).

Finally, we can report that successful waste management has a significant impact in the economy transition. So we can replace the sentence “waste as a problem” to “waste as a resource for new applications.” According to the characteristics of our investigated glass/ceramic composites, they can be successfully used in highly advanced applications with low-cost production methods. It can be used in optical lenses, laser optics, and electronic and magnetic sensor applications. They can be also used in the construction of materials as a substitute to other ingredients and in radiation shielding applications (Tyagi et al. 2020; Aygün et al. 2021).

## Conclusions

In an attempt of disposing and recycling the revealed industrial glass wastes, new advanced glass/ceramics composites were successfully produced. Promising Cr_2_O_3_/Fe_2_O_3_/LSG composites with various ceramics content (0–30 wt.%) have been produced by the low-cost pressureless sintering method. Different characteristics of the obtained composites such as phase composition, microstructure, and optical and magnetic properties were evaluated. Results indicated that parent LSG extracted from glass manufacturing industrial wastes has been successfully converted into promising advanced products for optoelectronic applications. The addition of chromia and hematite together to the glass waste had a significant effect on improving the morphological properties and optical and magnetic behaviors of the parent LSG material. Composite with the highest ceramic content of 30% Cr_2_O_3_/Fe_2_O_3_ has recorded the highest optical absorbance of 90%, the lowest and best band gap energy of 1.68 ev, and the highest refractive index of 2.85. Also, it has attained the best magnetic behavior with the highest saturation magnetization of 139.700 × 10^−2^A m^2^ kg^−1^ and coercivity of 190.0 Oe. Accordingly, our produced glass/ceramic composites can be successfully used as highly efficient optical and magnetic products for smart/advanced applications.

## Data Availability

The datasets generated during and/or analyzed during the current study are available from the corresponding author on reasonable request (E.M).
